# The different effects of molybdate on Hg(II) bio-methylation in aerobic and anaerobic bacteria

**DOI:** 10.3389/fmicb.2024.1376844

**Published:** 2024-07-02

**Authors:** Lanjing Wang, Hang Liu, Feng Wang, Yongmin Wang, Yuping Xiang, Yongyi Chen, Jiwu Wang, Dingyong Wang, Hong Shen

**Affiliations:** ^1^College of Resources and Environment, Southwest University, Chongqing, China; ^2^Research Center of Biology, Southwest University, Chongqing, China; ^3^Chongqing Engineering Research Center for Agricultural Non-Point Source Pollution Control, Chongqing, China

**Keywords:** mercury methylation, biotransformation, facultative anaerobic bacteria, sulfate-reducing bacteria, molybdate

## Abstract

In nature, methylmercury (MeHg) is primarily generated through microbial metabolism, and the ability of bacteria to methylate Hg(II) depends on both bacterial properties and environmental factors. It is widely known that, as a metabolic analog, molybdate can inhibit the sulfate reduction process and affect the growth and methylation of sulfate-reducing bacteria (SRB). However, after it enters the cell, molybdate can be involved in various intracellular metabolic pathways as a molybdenum cofactor; whether fluctuations in its concentration affect the growth and methylation of aerobic mercury methylating strains remains unknown. To address this gap, aerobic γ-Proteobacteria strains *Raoultella terrigena* TGRB3 (B3) and *Pseudomonas putida* TGRB4 (B4), as well as an obligate anaerobic δ-Proteobacteria strain of the SRB *Desulfomicrobium escambiense* CGMCC 1.3481 (DE), were used as experimental strains. The growth and methylation ability of each strain were analyzed under conditions of 500 ng·L^−1^ Hg(II), 0 and 21% of oxygen, and 0, 0.25, 0.50, and 1 mM of MoO_4_^2−^. In addition, in order to explore the metabolic specificity of aerobic strains, transcriptomic data of the facultative mercury-methylated strain B3 were further analyzed in an aerobic mercuric environment. The results indicated that: (a) molybdate significantly inhibited the growth of DE, while B3 and B4 exhibited normal growth. (b) Under anaerobic conditions, in DE, the MeHg content decreased significantly with increasing molybdate concentration, while in B3, MeHg production was unaffected. Furthermore, under aerobic conditions, the MeHg productions of B3 and B4 were not influenced by the molybdate concentration. (c) The transcriptomic analysis showed several genes that were annotated as members of the molybdenum oxidoreductase family of B3 and that exhibited significant differential expression. These findings suggest that the differential expression of molybdenum-binding proteins might be related to their involvement in energy metabolism pathways that utilize nitrate and dimethyl sulfoxide as electron acceptors. Aerobic bacteria, such as B3 and B4, might possess distinct Hg(II) biotransformation pathways from anaerobic SRB, rendering their growth and biomethylation abilities unaffected by molybdate.

## 1 Introduction

Methylmercury (MeHg) is the most toxic form of mercury (Li and Cai, 2013; Yang et al., 2020), which displays biotoxicity in bioaccumulation and biomagnification throughout the food chain. In the natural environment, the non-biological methylation rates of mercury are negligible (Berman and Bartha, 1986; Ullrich et al., 2001; Barkay et al., 2003; Raposo et al., 2008). Current research on the biological production and accumulation of MeHg focuses on anaerobic microorganisms possessing an inherent Hg biomethylation ability, which are involved in the transformation of the form of mercury from divalent mercury to MeHg (Parks et al., 2013; Tang et al., 2020). Mainly the δ-Proteobacteria class consisting of sulfate-reducing bacteria (SRB) (Compeau and Bartha, 1985; Gilmour and Henry, 1991), iron-reducing bacteria (Fleming et al., 2006; Kerin et al., 2006) and methanogens (Jensen and Jernelov, 1969; Gilmour et al., 2013) are included. Research on SRB methylation in the water level fluctuation zone (WLFZ) of the Three Gorges Reservoir, China, showed that SRB are not the predominant mercury methylators; instead, aerobic or facultative anaerobic microbial populations assume a pivotal role in biotic Hg(II) bio-methylation (Chen et al., 2016). Aerobic and/or facultative aerobic bacterial strains of the γ-Proteobacteria class, such as *Raoultella terrigena* strain TGRB3 (B3) and *Pseudomonas putida* strain TGRB4 (B4) (Mi et al., 2019) were screened and identified; it was found that both strains are capable of Hg bio-methylation under aerobic conditions. Among them, bacterium B3 is also capable of Hg bio-methylation under anaerobic conditions (Feng et al., 2022). The *hgcA/B* gene has been identified as a key gene for mercury methylation (Parks et al., 2013), MeHg production was detected in some habitats, but no *hgcA/B* gene was found under aerobic conditions (Podar et al., 2015); we also found that *hgcA/B* genes were absent in both B3 and B4 strains (Xiang et al., 2020). This different distribution suggests that there may be another unidentified metabolic pathway of Hg(II) bio-methylation where the mercury methylation capacity is independent of *hgcA/B* genes.

The Hg(II) biomethylation ability of bacteria depends on the properties of the strain rather than on the species of bacteria or the type of metabolism (Ranchou-Peyruse et al., 2009; Bridou et al., 2011). Moreover, this process is notably sensitive to environmental variables such as pH and salinity (Podar et al., 2015). Molybdenum, a crucial biological trace element, and molybdate were absorbed by organisms through either the high-affinity ModABC system (Xia et al., 2018) or the low-affinity CysPTWA (SulT) sulfate-thiosulfate permease (Aguilar-Barajas et al., 2011). Absorbed molybdenum associates with molybdopterin to generate molybdenum cofactors, or it combines with iron–sulfur clusters to form iron-molybdenum cofactors (Schwarz et al., 2009). These cofactors play a fundamental role in the facilitation of electron transfer during intracellular bio-oxidation processes (Iobbi-Nivol and Leimkuehler, 2013); they also catalyze conversions in carbon (Huang et al., 2022), nitrogen (Seefeldt et al., 2009), and sulfur compound metabolisms (Kisker et al., 1997). Because of its electron transfer capacity and involvement in redox reactions (Hille, 2013), molybdate might perturb the cellular sulfur metabolism, thereby influencing bacterial growth or methylation efficiency (Fleming et al., 2006; Thomas et al., 2019). In anaerobic Hg(II) bio-methylation microorganisms, molybdate can affect both SRB growth and Hg(II) bio-methylation capacity as a sulfate reduction metabolic inhibitor after entering the cell (Barkay and Wagner-Dobler, 2005; Yu and Barkay, 2022). However, information on molybdate in aerobic bacterial Hg(II) bio-methylation is limited. With DE as anaerobic control and B4 and B3 as aerobic control, an experiment was carried out to explore the responses of bacterial Hg(II) bio-methylation to molybdate, oxygen content, and different bacterial species. Moreover, transcriptomic sequencing was carried out to analyze gene expression in the aerobic B3. The effects of sodium molybdate at varying concentrations are tested on bacterial growth and Hg(II) bio-methylation efficiency, and the involvement of molybdenum-containing proteins/enzymes in the Hg (II) reduction process is evaluated. The results promote the available understanding of the mercury biogeochemical cycle in the soil of the seasonal WLFZ in the Three Gorges Reservoir area.

## 2 Materials and methods

### 2.1 Experimental design of the effect of molybdate on bacteria

#### 2.1.1 Experimental strains and culture conditions

*Raoultella terrigena* TGRB3 (B3) (GenBank accession number: MK102091), which belongs to γ-Proteobacteria, was isolated from the soil of the WLFZ in the Three Gorges Reservoir area (E108°12′3″, N30°24′36″) (Mi et al., 2019). B3 is capable of Hg(II) bio-methylation under both aerobic and anaerobic conditions. *Pseudomonas putida* TGRB4 (B4) (GenBank accession number: MF996382), which also belongs to γ-Proteobacteria, was isolated from the sediment soil of the Three Gorges Reservoir area (E108°12′3″, N30°24′36″) (Xiang et al., 2020). B4 is an obligate aerobic Hg(II) bio-methylation strain. For the Hg(II) bio-methylation experiment, B3 and B4 were cultured in the KB medium following activation in the LB medium (Cao et al., 2021). *Desulfomicrobium escambiense* CGMCC 1.3481 (DE), which is an obligate anaerobic mercury-methylated strain, was provided by the Research Center for Eco-Environmental Sciences at the Chinese Academy of Sciences. DE was cultured using modified DSMZ medium 63, consisting of FeSO_4_ and sodium acetate as the electron acceptor and donor, respectively, as described previously (Zhi et al., 2019). Culture followed the GB/T14643.5–2009 national standard for activation and cultivation (George et al., 2008).

#### 2.1.2 Oxygen concentration conditions

The experiment adopted completely anaerobic (0% oxygen) and aerobic (21% oxygen) oxygen concentration conditions. In the complete anaerobic condition, 150 mL of DSMZ medium was added to 250 mL borosilicate glass bottles. Nitrogen of high purity was injected at a rate of 60.0 mL·min^−1^ for 5 min to effectively remove oxygen by the Hungate copper column method (Hungate, 1969; Miller and Wolin, 1974). Additionally, resazurin was added as an aerobic color indicator. The strains B3 and B4 were cultured in KB medium, while DE was cultured in modified DSMZ medium 63 to ensure optimal bacterial growth. Non-inoculated blank media were utilized as controls for each treatment. For the aerobic system, 150 mL of KB medium was added to a 250 mL borosilicate glass bottle, and the gas inside the bottle was replaced with a high-purity gas mixture containing 21% oxygen and 79% nitrogen, flowing at a rate of 60.0 mL·min^−1^ for 5 min. B3 and B4 were cultured in KB medium, using a non-inoculated medium as blank controls. All treatments were cultured in a constant temperature incubator at 30°C at a uniform speed of 120 r·min^−1^. After 72 h, the oxygen content of 21% oxygen treatment was approximately 7.5 ± 0.5 mg·L^−1^, while the oxygen content of anaerobic treatment was 0.36 ± 0.1 mg·L^−1^ (Cao et al., 2021; Feng et al., 2022).

#### 2.1.3 Effects of molybdate on bacterial growth and Hg bio-methylation efficiency

**Figure 1:**
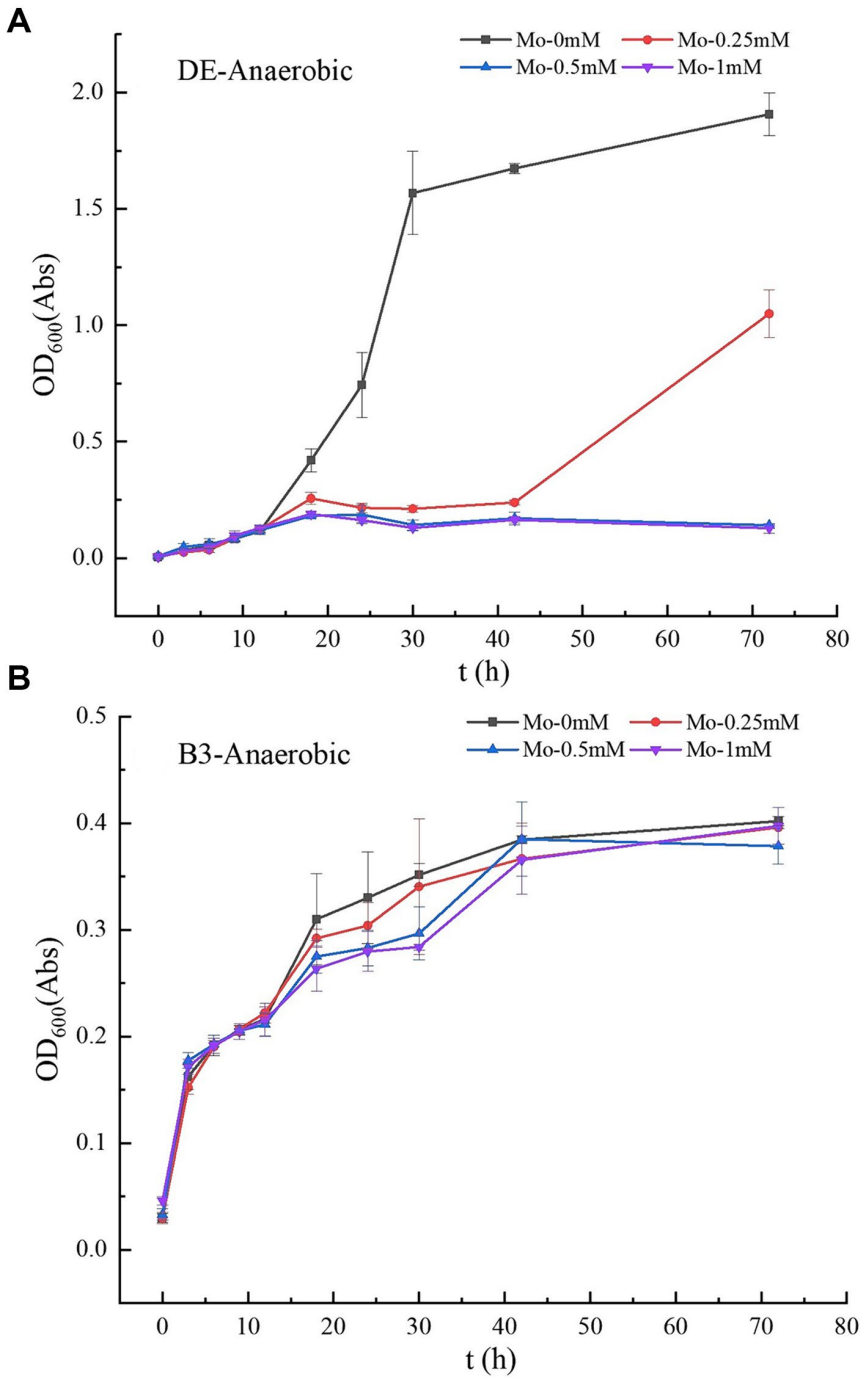
**(A–B)** Growth curves of strains DE and B3 under anaerobic conditions with different molybdate concentrations. DE represents *Desulfomicrobium escambiense* CGMCC 1.3481 strain and B3 represents *Raoultella terrigena* TGRB3 strain.

**Figure 2:**
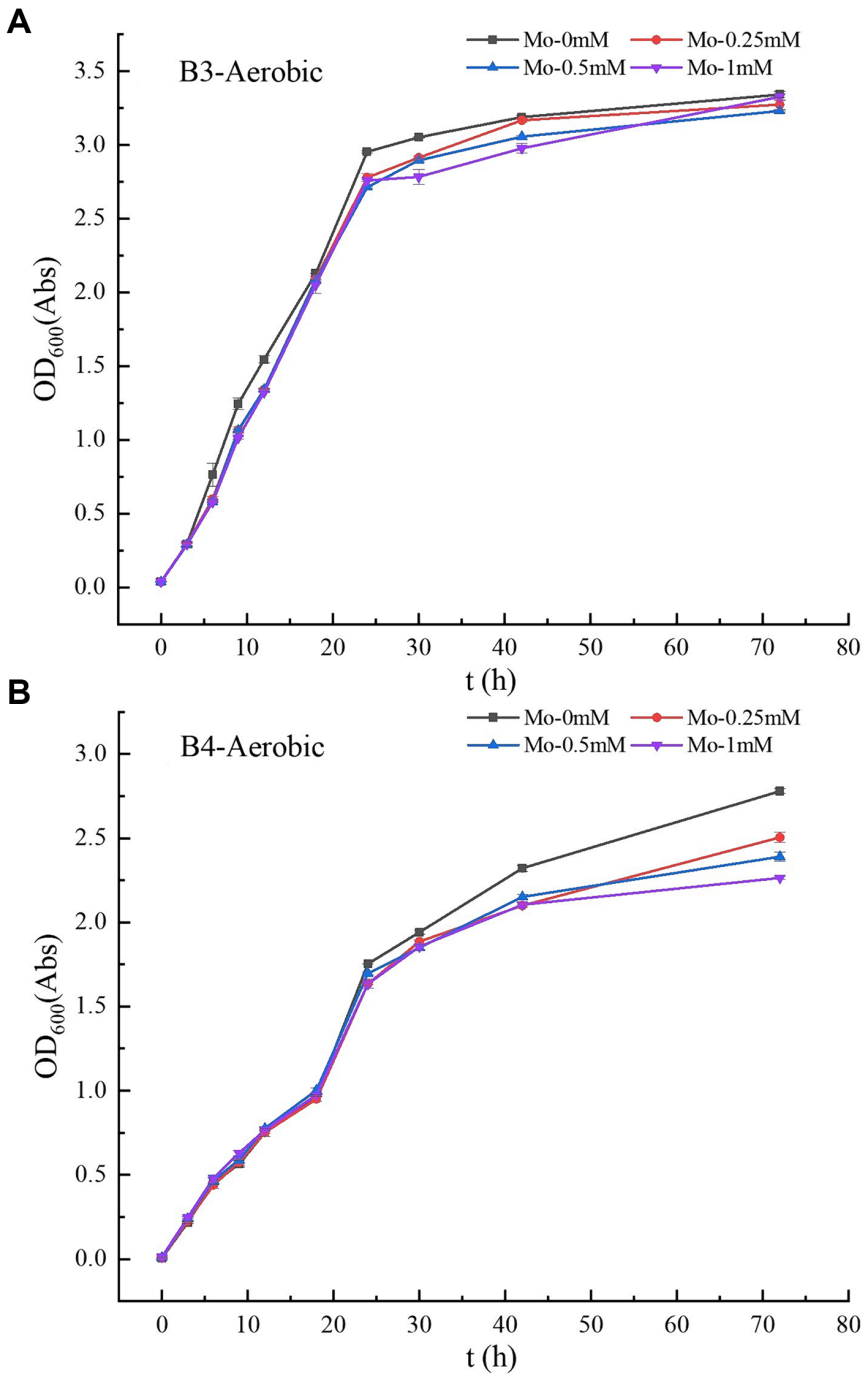
**(A–B)** Growth curves of strains B3 and B4 under aerobic conditions with different molybdate concentrations. B3 represents *Raoultella terrigena* TGRB3 strain and B4 represents *Pseudomonas putida* TGRB4 strain.

Under both anaerobic and aerobic conditions, four levels of molybdate ion (MoO_4_^2−^) concentrations [released from Na_2_MO_4_ (analytical grade)] of 0, 0.25, 0.50, and 1 mM were tested (Fleming et al., 2006). The medium for each bacterial culture was the same as described in Section 2.1.2, and cells were harvested during the mid-exponential phase (OD_600_ = 0.7–0.8). After the cells had been harvested, they were washed three times with sterile Milli-Q water, then centrifuged (2,430 *g*, 25°C, 5 min), and finally suspended in sterile Milli-Q water and mixed into the culture system (Feng et al., 2022). A bacteria concentration of approximately 10^7^ cells·mL^−1^ was guaranteed, and bacteria were cultured under a constant temperature of 30°C. Finally, samples were collected at 0, 3, 6, 9, 12, 18, 24, 30, 42, and 72 h. The sterile medium served as a blank control for each treatment. In addition, molybdate and mercury solution were added to the culture bottle using disposable sterile syringes to eliminate bubbles and then added to ensure anaerobic conditions. In this experiment, the bacterial density was measured using spectrophotometry (OD_600_), and the maximum growth values and significant differences between treatments are shown in Table 1. In addition, the growth curves of each strain under different oxygen and molybdenum concentrations are shown in Figures 1, 2. All treatments were performed in triplicate, and the average value was used for analysis. The details of each experimental treatment are listed in Supplementary Table S1.

**Table 1:** Maximum OD_600_ (Abs) of bacteria under different conditions of molybdate concentration in 72 h.

Molybdate (mM)	B3		B4		DE	
	Anaerobic	Aerobic	Anaerobic	Aerobic	Anaerobic	Aerobic
0	0.40 ± 0.01 aC	3.34 ± 0.02 *aA	–	2.78 ± 0.02 aB	1.91 ± 0.10 aA	–
0.25	0.40 ± 0 aC	3.27 ± 0.03 *aA	–	2.51 ± 0.03 abB	1.05 ± 0.10 bB	–
0.50	0.39 ± 0.03 aC	3.23 ± 0.01 *aA	–	2.39 ± 0.03 abB	0.19 ± 0.04 cC	–
1.0	0.40 ± 0.02 aC	3.33 ± 0.02 *aA	–	2.27 ± 0.01 bB	0.19 ± 0.01 cC	–

The lowercase letters indicate differences in molybdate concentrations for the same strain at the same oxygen concentration (*p* < 0.05). The capital letters indicate differences for different strains at the same oxygen concentrations (*p* < 0.05). *represents the difference of the same strain in different molybdate concentrations under different oxygen concentrations (*p* < 0.05).

Based on molybdate experiments, HgCl_2_ (analytical grade) was used as an Hg(II) donor. The final concentration of experimental Hg(II) was set to 500 ng·L^−1^ (Cao et al., 2021), and treatment without Hg(II) addition was conducted as a control. The bacterial inoculation method and culture conditions were the same as mentioned above, and samples were collected at 0, 6, 12, 18, 30, 42, and 72 h. At harvest, the samples were divided into two portions: one sub-sample was used for bacterial growth determination; the other sub-samples were stopped by acidification with HCl to a final concentration of 0.5% (v/v), followed by storage at −20°C until total Hg and MeHg analysis. The detailed information about experimental treatments and their corresponding codes are shown in Supplementary Table S2.

### 2.2 TGRB3 transcriptome sequencing experiment

#### 2.2.1 Experimental strains and culture conditions

*Raoultella terrigena* TGRB3 (B3) (GenBank accession number: MK102091), as well as the bacterial culture method and harvesting procedures, were consistent with the information provided in Section 2.1.3.

#### 2.2.2 Transcriptome sequencing and data processing

To ensure transcriptional differences between samples, the experimental group was exposed to exogenous Hg(II) at a final concentration of 500 ng·L^−1^, while the control group was maintained without the addition of exogenous mercury (0 ng·L^−1^). Bacteria were harvested at 3, 9, and 24 h during the experiment, and a total of 12 samples were collected. The detailed sample descriptions are provided in Supplementary Table S3. Bacterial total RNA was extracted using the rapid bacterial RNA extraction kit (AiDLab). Sequencing was performed on the Illumina HiSeq platform, and transcriptome sequencing data were completed and returned by Shanghai LingEn Company. The original transcriptome data have been uploaded to the Sequence Read Archive (SRA) in the NCBI database under the accession number PRJNA1111264. After that, transcriptomic sequencing raw data were processed using Trimmomatic software to remove adapter sequences and low-quality reads from the ends. Subsequently, filtered high-quality sequences were aligned and analyzed against a reference genome using Rockhopper software, enabling their mapping to the annotated genome. The expression levels of individual genes were quantified and normalized to reads per kilobase per million mapped reads (RPKM) for inter-sample differential expression calculations. RPKM was calculated as follows:


$$\mathrm{RPKM}=\frac{\mathrm{total}\;\mathrm{exon}\;\mathrm{reads}}{\mathrm{mapped}\;\mathrm{reads}\left(\mathrm{millions}\right)\times \mathrm{exon}\;\mathrm{length}\left(KB\right)}$$

Finally, differentially expressed genes (DEGs) were identified based on stringent criteria (FDR ≤ 0.05 and |log2(FC)| ≥ 1), and DEG sequences were aligned to databases such as the NR, GO, COG, KEGG, and Swiss-Prot (e-value <10^−5^) to obtain crucial protein annotation information for these genes (Kanehisa et al., 2016). The GO analysis and KEGG pathway enrichment analysis were performed using Goatools^1^ and the KOBAS^2^ online platform, respectively (Klopfenstein et al., 2018; Bu et al., 2021).

### 2.3 Mercury detection and quality control

MeHg was quantified using distillation-ethylation cold atomic fluorescence spectrometry (GC-CVAFS) on a Tekran 2,500 Mercury Meter from Brooks Rand Ltd. The total Hg content was measured through double gold amalgam-cold atomic fluorescence spectrometry (Liang et al., 1994; Feng et al., 1998; Yan et al., 2003). Quality assurance and quality control were conducted based on the utilization of parallel samples and blank controls for all experimental samples during the analysis process. For MeHg determination, quality control included both blank distillation and spike recovery, resulting in a recovery rate of 80–120% and a parallel sample coefficient of variation of less than 10%. Prior to total Hg determination, the enrichment system was thoroughly purified using 5% concentrated HNO_3_ and 1–2 mL of SnCl_2_ to eliminate potential sources of contamination. Sample analysis was only performed when the mercury content in the blank sample was below 5 pg., guaranteeing minimal interference. All experimental reagents were of superior purity, and borosilicate glassware was soaked in 25% (v/v) nitric acid for at least 24 h, followed by rinsing with ultrapure water (Milli-Q, 18 Ω·CM); finally, glassware was purified by heating at 500°C for 30 min in a muffle furnace to eliminate mercury contamination.

### 2.4 Data analyses

The data (mean ± SD; *n* = 3) were subjected to analysis of variance. Significant differences between treatment means were compared using Tukey’s honestly significant difference (HSD) test (*p* ≤ 0.05) and independent-samples t-test in SPSS Statistics 26 (NY, USA). All figures are plotted with Origin 2017 (OriginLab, USA).

## 3 Results and discussion

### 3.1 Effects of molybdate on bacterial growth

#### 3.1.1 Bacterial growth under anaerobic conditions

Molybdate concentration significantly affected the growth of anaerobic mercury-methylated *Desulfomicrobium escambiense* CGMCC 1.3481 (DE) (Table 1; Figure 1A). After 72 h of culture, compared to the maximum OD_600_ value of 1.91 ± 0.1 Abs in the treatment without molybdenum, the growth capacity of bacteria decreased by 45, 90, and 90% at 0.25, 0.5, and 1 mM molybdenum concentrations, respectively. Under a molybdenum concentration of 0.5 mM, the growth of DE bacteria was almost completely inhibited. In accordance with previous research (Newport and Nedwell, 1988), the results showed that adenosine phosphosulfate formed when molybdate entered the cells. Adenosine phosphosulfate interfered with the sulfate reduction process of bacteria and exerted a strong inhibitory effect on the growth of DE (Barkay and Wagner-Dobler, 2005; Patidar and Tare, 2005; Correia et al., 2012). However, the treatment of *Raoultella terrigena* TGRB3 (B3) achieved no significant difference in the effect of changes in molybdate concentration on the growth capacity of this bacterial strain (Figure 1B). As B4—a strictly aerobic bacterium—cannot grow normally under anaerobic conditions, the growth and subsequent methylation of strain B4 were not analyzed. These results suggest that the two classes of bacteria adopted different respiratory electron transport chains. In other words, anaerobic respiration of B3 bacteria might adopt a non-sulfate metabolic pathway.

#### 3.1.2 Bacterial growth under aerobic conditions

The experimental results showed that molybdate concentration did not affect the growth of B3 (Table 1; Figure 2A), and OD_600_ values of 0.39–0.40 Abs were maintained among the four molybdate treatments. However, in the treatment of strain B4, the addition of 1 mM molybdenum significantly inhibited the number of bacteria compared to the treatment without molybdenum (Figure 2B); this result was not observed in the 0.25 and 0.5 mM treatment groups. Moreover, under the same oxygen concentration, the growth ability of B3 was significantly better than that of strain B4. Furthermore, under aerobic conditions, the growth of B3 was significantly better than under anaerobic conditions (Table 1). B4 could not grow under anaerobic conditions, while DE could not grow under aerobic conditions.

The aforementioned experimental results indicate that changes in molybdate concentration lead to significant inhibitory effects on the anaerobic growth of SRB but did not significantly impact the growth of the B3 strain. This result is consistent with previous research findings showing that SRB acquire energy through the reduction of sulfate; molybdate acts as a competitive inhibitor of sulfate, leading to targeted “energy uncoupling” and inhibiting the growth of SRB (Wilson and Bandurski, 1958; Fleming et al., 2006). Interestingly, the experimental results also suggest that under aerobic conditions, strains within the same γ-Proteobacteria class utilize different bio-oxidation pathways: Molybdate could not impact the growth of the B3 strain, while the growth of B4 was significantly affected by the molybdate concentration. This result suggests that bacteria of the γ-Proteobacteria class might possess multiple bio-oxidation pathways. Among them, certain strains do not rely on sulfur metabolism (e.g., B3), while others utilize alternative sulfur metabolism pathways (e.g., B4). The choice of metabolic pathways may be determined by the distinct biological attributes of different bacteria.

### 3.2 Efficiency of bacterial Hg(II) bio-methylation

#### 3.2.1 Analysis of Hg(II) bio-methylation by DE and B3 under anaerobic conditions

**Figure 3:**
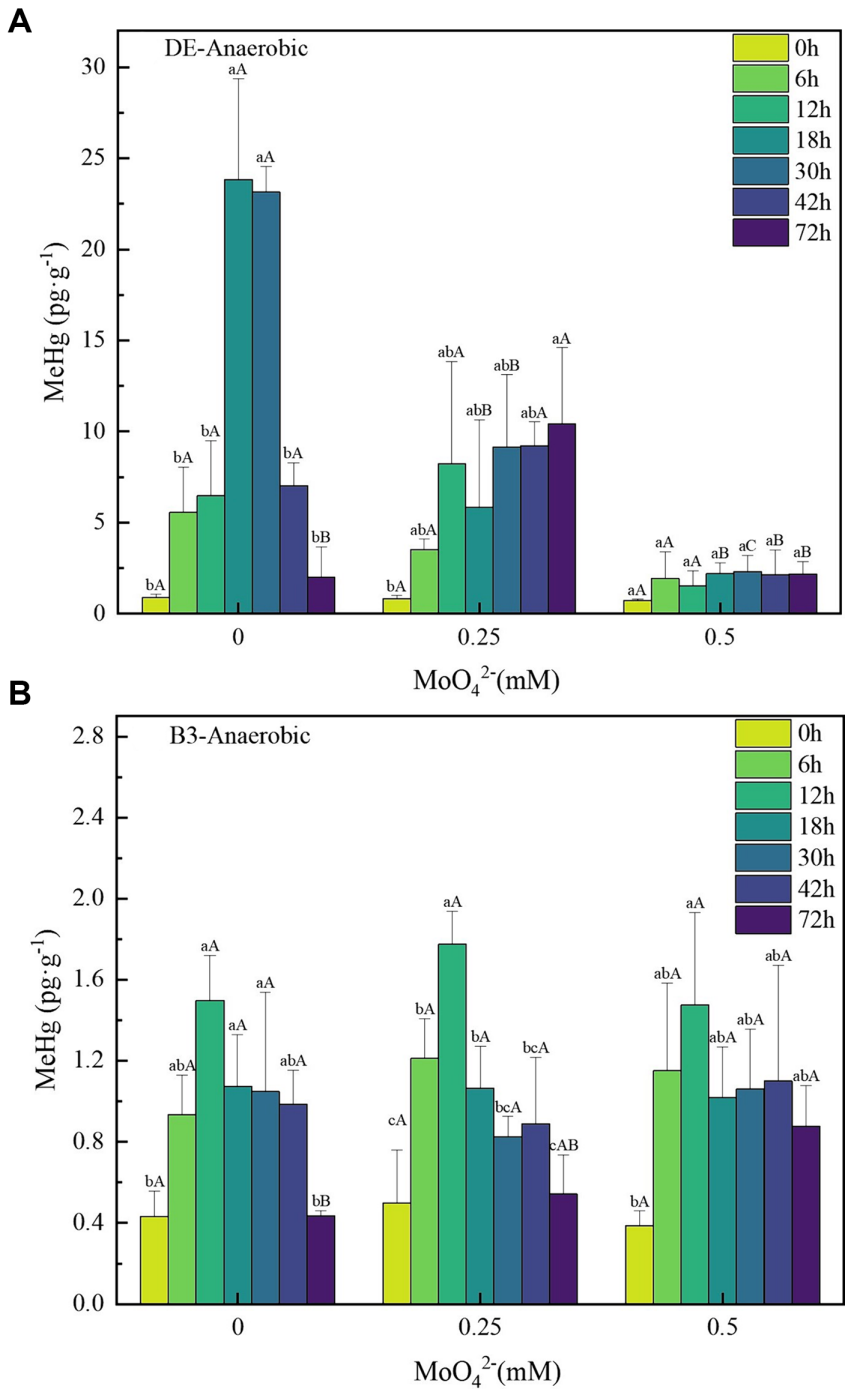
**(A–B)** Changes in unit methylmercury content of strains DE and B3 under anaerobic conditions with different molybdate concentrations. Lowercase letters indicate the significant differences in MeHg content within 72 h at the same molybdate concentration (*p* < 0.05); capital letters indicate significant differences in MeHg content under different molybdate concentrations at the same time (*p* < 0.05). DE represents *Desulfomicrobium escambiense* CGMCC 1.3481 strain and B3 represents *Raoultella terrigena* TGRB3 strain.

Under anaerobic conditions with a Hg(II) concentration of 500 ng·L^−1^, in the DE bacteria treatment without added molybdate, the peak of MeHg content was reached at 18 h. The maximum net concentration of methylmercury ([MeHg]_max_) was 22.94 ± 5.7 pg·g^−1^ (Figure 3A). The concentration of MeHg showed a regular decrease with increasing MoO_4_^2−^ concentration. The [MeHg]_max_ of the 0.25 and 0.5 mM molybdate treatments were 9.59 ± 4.03 pg·g^−1^ at 72 h and 1.58 ± 0.87 pg·g^−1^ at 30 h, respectively. Compared to the DE-Hg500-Mo0 treatment, the maximum Hg(II) bio-methylation concentrations decreased by 58.21 and 81.11%, respectively.

In contrast, the MeHg content of the facultative bacterial strain B3 reached its peak earlier than that of DE, but its methylation capacity was much lower than that of DE, and its [MeHg]_max_ was 1.07 ± 0.28 pg·g^−1^ (Figure 3B). With increasing MoO_4_^2−^ concentration, the MeHg content of B3 did not show regular changes. Moreover, the deviation of the MeHg content among different concentration gradients was less than 0.2 pg·g^−1^, the decrease of [MeHg]_max_ was less than 15%, and no significant difference was found.

Consistent with the results of previous studies, molybdate, as a metabolic inhibitor of SRB, can affect their Hg(II) biomethylation ability. This inhibitory effect is gradually strengthened with increasing molybdate concentration (Fleming et al., 2006). This conclusion is supported by the results of the molybdate inhibition test against strain DE in this experiment. The effect of molybdate on sulfate reduction resulted in the obstruction of cell energy acquisition, which affected its growth and eventually led to the loss of mercury methylation ability at a concentration of 1 mM molybdate. In contrast, the ability of Hg(II) bio-methylation in B3 treatments was not significantly impacted by molybdate. Although it is not possible to predict the specific metabolic pathway adopted by bacteria B3, the fact that the metabolic pathways of bacteria B3 and strain DE are different is at least confirmed.

#### 3.2.2 Analysis of Hg(II) bio-methylation by B3 and B4 under aerobic conditions

**Figure 4:**
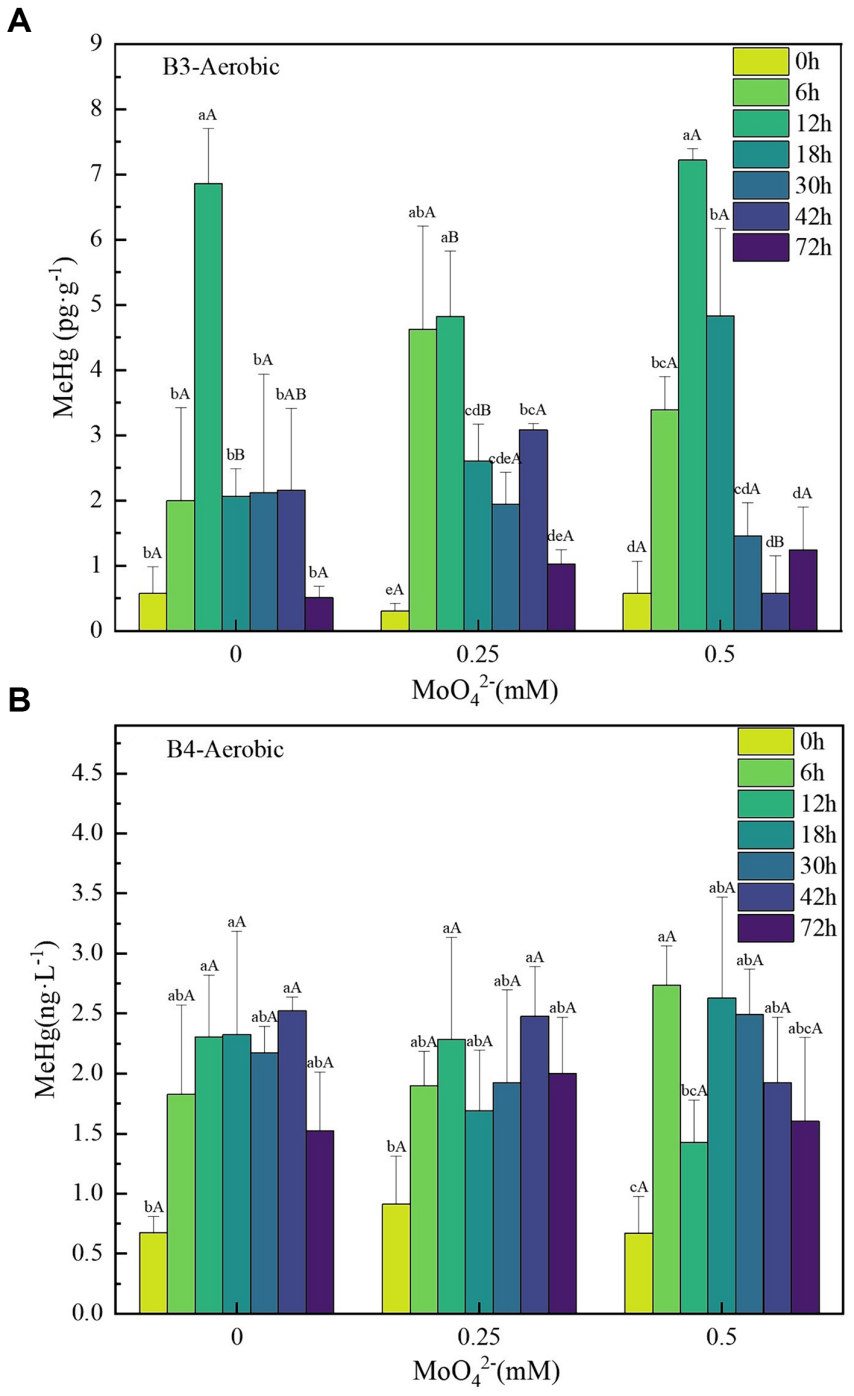
**(A–B)** Changes in unit methylmercury content of strains B3 and B4 under aerobic conditions with different molybdate concentrations. Lowercase letters indicate significant differences in MeHg content within 72 h at the same molybdate concentration (*p* < 0.05); capital letters indicate the significant difference in MeHg content under different molybdate concentrations at the same time (*p* < 0.05). B3 represents *Raoultella terrigena* TGRB3 strain, and B4 represents *Pseudomonas putida* TGRB4 strain.

Based on previous tests, the Hg(II) concentration of 500 ng·L^−1^ is well within the tolerance range of B3 and B4 bacteria and does not affect the growth of both (Cao et al., 2021; Feng et al., 2022). Under the 500 ng·L^−1^ Hg(II) condition, the [MeHg]_max_ in the B3 and B4 bacterial treatments without added molybdate treatments were 6.28 ± 0.46 and 1.85 ± 0.19 pg·g^−1^, respectively (Figures 4A,B); however, their [MeHg]_max_ decreased to 4.51 ± 1.0 and 1.56 ± 0.82 pg·g^−1^, respectively, at a MoO_4_^2−^ concentration of 0.25 mM. This trend was not stable and their [MeHg]_max_ increased to 6.65 ± 0.32 and 2.07 ± 0.06 pg·g^−1^, respectively, at 0.5 mM concentration. No significant difference was found in the effect of MoO_4_^2−^ concentration on MeHg concentration of B3 and B4 strains under aerobic conditions.

The comprehensive experimental results indicate that the Hg(II) bio-methylation efficiency of δ-Proteobacteria class strains might be significantly negatively correlated with molybdate concentrations. This correlation stems from the interference of molybdate with the bacterial sulfur metabolism, leading to the inhibition of bacterial growth and consequently affecting Hg(II) bio-methylation (Fleming et al., 2006). Because of their lack of sulfur metabolism, the bacterial growth of γ-Proteobacteria could not be inhibited by molybdate, and the effect on Hg(II) bio-methylation was not significant. Further analysis from a mechanistic perspective shows that the *hgcA/B* gene can be found in most anaerobic mercury methylation microorganisms, especially SRB. Research has shown that there is a strong covariance between mercury methylation and sulfate reduction in both time and space, indicating the critical contribution of SRB to mercury methylation (Regnell and Watras, 2019). At the same time, an increasing number of studies have detailed the contribution of aerobic microorganisms to methylation (Jones et al., 2019; Zhang et al., 2021). In this study, the mercury methylation ability of B3 and B4 bacteria was also confirmed, but no evidence of *hgcA/B* gene was found in B3 and B4 strains; moreover, no reports of the presence of *hgcA/B* were found in aerobic mercury methylation microorganisms. This suggests that the evolutionary migration of *hgcA/B* genes is also related to metabolism.

### 3.3 Transcriptome data analysis of B3 strains under Hg(II) stress conditions

#### 3.3.1 Analysis of DEGs under Hg(II) stress conditions

**Figure 5:**
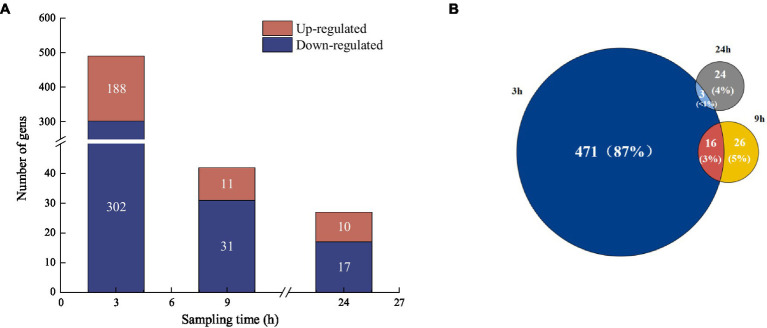
**(A)** Analysis of differentially expressed genes at different sampling times compared to control groups. **(B)** Venn diagram analysis of the proportion of differentially expressed genes at different sampling times.

To examine the metabolic type of mercury methylation in *Raoultella terrigena* TGRB3 (B3), DEGs were analyzed under 500 ng·L^−1^ Hg(II) conditions. In the comparison between the control group and the experimental group at 3, 9, and 24 h, totals of 490, 42, and 27 DEGs were identified, respectively; among them, 188, 11, and 10 genes were upregulated, respectively, while 302, 31, and 17 genes were downregulated, respectively (Figure 5A). A comparison of unique DEGs in each group is shown in Figure 5B. Notably, the highest number of DEGs (471) was observed at 3 h. These results suggest that the bacterial response to environmental Hg (II) toxicity might occur in the initial stage of exposure to environmental Hg (II) conditions. At 9 h, however, the number of DEGs decreased significantly to 26, which could be explained by bacterial adaptability and their regulatory ability to the environment.

#### 3.3.2 Analysis of DEGs in bacterial metabolism under Hg(II) conditions

The abovementioned criteria (FDR ≤ 0.05 and |log2(FC)| ≥ 1) were used to screen for DEGs related to bacterial metabolism (Table 2). Under 500 ng·L^−1^ Hg(II) and aerobic conditions, the *cydB* gene associated with aerobic respiration was significantly differentially expressed. This gene was annotated to a subunit of the cytochrome bd-I complex located at the terminal oxidase of the *Escherichia coli* respiratory chain and assumes a pivotal role in the NADH decomposition metabolism (Hamed et al., 2020). In addition, a series of genes related to cellular anaerobic metabolism (i.e., *frdc*, *frdD*, *dcuA*, *dcuB*, and *napA*) were significantly upregulated, which were annotated to engage in nitrate or nitrite reduction. The results were in accordance with the results of *E. coli* under aerobic conditions (Ingledew and Poole, 1984).

**Table 2:** Cell respiration and genes related to metabolism.

Symbol	Gene ID	KEGG ID	Gene name	Gene description	Log(2)FC
(3 h) Hg500 vs. Hg0	B-1000711	K00247	*frdD*	Fumarate reductase subunit D	1.16
	B-1000712	K00246	*frdC*	Fumarate reductase subunit C	1.16
	B-1000695	K07791	*dcuA*	Anaerobic C4-dicarboxylate transporter	1.28
	B-1000676	K07792	*dcuB*	Anaerobic C4-dicarboxylate transporter	1.18
	B-1003236	K00373	*narJ*	Nitrate reductase molybdenum cofactor assembly chaperone	−1.47
	B-1003237	K00371	*narH*	nitrate reductase subunit beta	−1.44
	B-1003238	K00370	*narG*	Nitrate reductase subunit alpha	−1.38
	B-1003585	K02567	*napA*	Nitrate reductase catalytic subunit	1.0
	B-1001725	K00247	*cydB*	Cytochrome bd-I ubiquinol oxidase subunit	1.0

Based on the identified DEGs, it can be speculated that facultative anaerobic bacteria (i.e., strain B3) undergo adaptive modifications because of the fluctuating oxygen availability in the soil of the WLFZ in the Three Gorges Reservoir (Cao et al., 2021). The adaptive process of multiple energy metabolic pathways could simultaneously or alternately utilize diverse energy sources and metabolic strategies (Ortiz et al., 2020). This result is similar to a previous study that found Bin 144 bacteria from the candidate phylum *Aminicenantes* in the Louis River watershed, northern Minnesota, also without the *hgcA/B* gene. The reason for this observed similarity is that it encodes the respiratory complex cytochrome C oxidase (*coxABCD*), suggesting that it has other respiratory functions and may be able to use these oxygenases to resist mercury stress under low oxygen conditions (Jones et al., 2019). Perhaps the respiratory and metabolic types of strain B3 in this study are similar to those of strain Bin 44, but further verification of this supposition is needed.

#### 3.3.3 Analysis of expressions of genes related to molybdenum

Analysis of the transcriptional data showed that certain genes related to molybdenum unexpectedly showed significant alterations (Table 3). These genes are involved in the nitrate reductase molybdenum cofactor assembly chaperone (*narJ*), the dimethyl sulfoxide reductase (DMSO) subunit, the selenate reductase subunit (*ygfK*), and the molybdate-dependent oxidoreductase FAD-binding subunit (*ygfM*). In addition, certain molybdenum enzyme families were involved in the DMSO family, the xanthine oxidase family, and the sulfite oxidase family (Iobbi-Nivol and Leimkuehler, 2013). Notably, DEGs belonging to both the DMSO and nar-type nitrate reductase groups were members of DMSO. These genes participate in the synthesis and consumption of dimethyl sulfide, with DMSO reductase forming an integral component of the bacterial respiratory chain. These genes could act as an alternate terminal reductase, thus playing a role in anaerobic respiration and energy conservation under anaerobic conditions (Wilson and Bandurski, 1958).

**Table 3:** Differentially expressed genes related to molybdenum.

Symbol	Gene ID	KEGG ID	Gene name	Gene description	Log(2)FC
(3 h) Hg500 vs. Hg0	B-1003236	K00373	*narJ*	Nitrate reductase molybdenum cofactor assembly chaperone	−1.47
	B-1004410	K12529	*ygfM*	Molybdopterin-dependent oxidoreductase FAD-binding subunit	1.11
(3–9 h) Hg500 vs. Hg0	B-1000970	K03831	*mogA*	Molybdopterin adenylyltransferase	−1.61
	B-1001751	K02020	*modA*	Molybdopterin adenylyltransferase	−1.32
	B-1004408	K12527	*ygfK*	Putative selenate reductase subunit	−1.54
	B-1001924	K07307	*dmsB*	Dimethyl sulfoxide reductase subunit B	−1.29
	B-1001925	K07308	*dmsC*	Dimethyl sulfoxide reductase anchor subunit	−1.29
	B-1004410	K12529	*YgfM*	Molybdopterin-dependent oxidoreductase FAD-binding subunit	1.56
(9–24 h) Hg500 vs. Hg0	B-1001924	K07307	*dmsB*	Dimethyl sulfoxide reductase subunit B	1.08

Based on the DEGs of Mo-related genes, it can be speculated that molybdenum enzymes might be associated with the metabolic pathways of the facultative anaerobic B3 strain. As an essential trace element, molybdenum plays a crucial role in bacterial growth and in the metabolism of cellular carbon, nitrogen, and sulfur (Kisker et al., 1997; Leimkuehler and Iobbi-Nivol, 2016). However, molybdate has been documented to function as a metabolic inhibitor in SRB, disrupting their sulfur metabolism and impeding both growth and methylation processes (Thomas et al., 2019). In non-sulfur-metabolizing Hg(II) bio-methylation bacteria, molybdenum-containing proteins or enzymes might assume an active role in alternative metabolic pathways to environmental mercury stress. Thereby, they play a role in the biological mercury stress defense. However, whether molybdate was involved in the Hg(II) reduction process cannot be identified, as current limitations in technical conditions prevent effective determination.

## 4 Conclusion

Anaerobic bacterial growth and Hg(II) bio-methylation of δ-Proteobacteria (i.e., strain DE) were significantly inhibited by molybdate because of a disruption of the bacterial sulfur metabolism. However, the aerobic bacteria of γ-Proteobacteria (i.e., strains B3 and B4) could grow normally in the molybdate-containing medium. These results suggest that B3 and B4 might be non-sulfuric in the bacterial electron transfer chain, as the metabolic pathway was not dependent on sulfate as an electron acceptor.

According to the temporal differences of the transcriptomic DEGs of B3, the bacterial response to environmental Hg(II) toxicity occurs in the initial stage of Hg(II) exposure. Therefore, the observed significant differences in molybdenum-containing proteins in B3 bacteria might be due to their participation in various intracellular biological oxidation processes. Examples of these processes are nitrate, nitrite, and DMSO energy metabolic pathways, as well as the role they play in electron transfer; ultimately, these processes mediate the defense mechanism of bacteria against mercury stress. In general, facultative B3 bacteria might have environmental oxygen adaptability and Hg(II) bio-methylation compensating metabolic pathways.

## Data availability statement

The datasets presented in this study can be found in online repositories. The names of the repository/repositories and accession number(s) can be found in the article/Supplementary material.

## Author contributions

LW: Conceptualization, Investigation, Writing – original draft. HL: Conceptualization, Formal analysis, Writing – review & editing. FW: Resources, Writing – review & editing. YW: Validation, Writing – review & editing. YX: Methodology, Writing – review & editing. YC: Resources, Writing – review & editing. JW: Data curation, Writing – review & editing. DW: Supervision, Writing – review & editing, Resources. HS: Project administration, Writing – review & editing, Funding acquisition.

## Footnotes

^1^ https://github.com/tanghaibao/Goatools

^2^ http://kobas.cbi.pku.edu.cn/home.do
