# Supplementary material for: The different effects of molybdate on Hg(II) bio-methylation in aerobic and anaerobic bacteria
Source: Front Microbiol. 2024 Jul 2;15:1376844. doi: 10.3389/fmicb.2024.1376844 (PMC11249568; doi:10.3389/fmicb.2024.1376844)
Supplement: Supplementary file 1 [file Table_1.DOCX]

Supplementary Material

Table S 1 Experimental treatment of the effect of different concentrations of molybdate on the growth of strains

| O_2_ Concentration (%) | Strains | Symbol Number | Note of Treatments |
| --- | --- | --- | --- |
| 21 | *Raoultella terrigena*  TGRB3 (B3) | B3-Mo0-21 | Inoculate with B3 bacteria, [MoO_4_^2-^]=0 mM, O_2_ Concentration (21%) |
|  |  | B3-Mo0.25-21 | Inoculate with B3 bacteria, [MoO_4_^2-^]=0.25 mM, O_2_ Concentration (21%) |
|  |  | B3-Mo0.5-21 | Inoculate with B3 bacteria, [MoO_4_^2-^]=0.5 mM, O_2_ Concentration (21%) |
|  |  | B3-Mo1-21 | Inoculate with B3 bacteria, [MoO_4_^2-^]=1 mM, O_2_ Concentration (21%) |
|  | *Pseudomonas putida* TGRB4 (B4) | B4-Mo0-21 | Inoculate with B4 bacteria, [MoO_4_^2-^]=0 mM, O_2_ Concentration (21%) |
|  |  | B4-Mo0.25-21 | Inoculate with B4 bacteria, [MoO_4_^2-^]=0.25 mM, O_2_ Concentration (21%) |
|  |  | B4-Mo0.5-21 | Inoculate with B4 bacteria, [MoO_4_^2-^]=0.5 mM, O_2_ Concentration (21%) |
|  |  | B4-Mo1-21 | Inoculate with B4 bacteria, [MoO_4_^2-^]=1 mM, O_2_ Concentration (21%) |
| 0 | *Raoultella terrigena*  TGRB3 (B3) | B3-Mo0-0 | Inoculate with B3 bacteria, [MoO_4_^2-^]=0 mM, O_2_ Concentration (0%) |
|  |  | B3-Mo0.25-0 | Inoculate with B3 bacteria, [MoO_4_^2-^]=0.25 mM, O_2_ Concentration (0%) |
|  |  | B3-Mo0.5-0 | Inoculate with B3 bacteria, [MoO_4_^2-^]=0.5 mM, O_2_ Concentration (0%) |
|  |  | B3-Mo1-0 | Inoculate with B3 bacteria, [MoO_4_^2-^]=1 mM, O_2_ Concentration (0%) |
|  | *Desulfomicrobium escambiense* CGMCC 1.3481 (DE) | DE-Mo0-0 | Inoculate with DE bacteria, [MoO_4_^2-^]=0 mM, O_2_ Concentration (0%) |
|  |  | DE-Mo0.25-0 | Inoculate with DE bacteria, [MoO_4_^2-^]=0.25 mM, O_2_ Concentration (0%) |
|  |  | DE-Mo0.5-0 | Inoculate with DE bacteria, [MoO_4_^2-^]=0.5 mM, O_2_ Concentration (0%) |
|  |  | DE-Mo1-0 | Inoculate with DE bacteria, [MoO_4_^2-^]=1 mM, O_2_ Concentration (0%) |

Table S 2 Experimental treatment of mercury methylation by molybdate under anaerobic and aerobic conditions

| O_2_ Concentration.(%) | Strains | Symbol Number | Note of Treatments |
| --- | --- | --- | --- |
| 21 | *Raoultella terrigena*  TGRB3 (B3) | B3-Hg500-Mo0-21 | Inoculate with B3 bacteria, [ MoO_4_^2-^]=0 mM，[Hg^2+^]=500 ng/L, O_2_ Concentration (21%) |
|  |  | B3- Hg500-Mo0.25-21 | Inoculate with B3 bacteria, [ MoO_4_^2-^]=0.25 mM，[Hg^2+^]=500 ng/L, O_2_ Concentration (21%) |
|  |  | B3 Hg500-Mo0.5-21 | Inoculate with B3 bacteria, [ MoO_4_^2-^]=0.5 mM，[Hg^2+^]=500 ng/L, O_2_ Concentration (21%) |
|  | *Pseudomonas putida* TGRB4 (B4) | B4-Hg500-Mo0-21 | Inoculate with B4 bacteria, [ MoO_4_^2-^]=0 mM，[Hg^2+^]=500 ng/L, O_2_ Concentration (21%) |
|  |  | B4-Hg500-Mo0.25-21 | Inoculate with B4 bacteria, [ MoO_4_^2-^]=0.25 mM，[Hg^2+^]=500 ng/L, O_2_ Concentration (21%) |
|  |  | B4-Hg500-Mo0.5-21 | Inoculate with B4 bacteria, [ MoO_4_^2-^]=0.5 mM，[Hg^2+^]=500 ng/L, O_2_ Concentration (21%) |
| 0 | *Raoultella terrigena*  TGRB3 (B3) | B3-Hg500-Mo0-0 | Inoculate with B3 bacteria, [ MoO_4_^2-^]=0 mM，[Hg^2+^]=500 ng/L, O_2_ Concentration (0%) |
|  |  | B3-Hg500-Mo0.25-0 | Inoculate with B3 bacteria, [ MoO_4_^2-^]=0.25 mM，[Hg^2+^]=500 ng/L, O_2_ Concentration (0%) |
|  |  | B3-Hg500-Mo0.5-0 | Inoculate with B3 bacteria, [ MoO_4_^2-^]=0.5 mM，[Hg^2+^]=500 ng/L, O_2_ Concentration (0%) |
|  | *Desulfomicrobium escambiense* CGMCC 1.3481 (DE) | DE-Hg500-Mo0-0 | Inoculate with DE bacteria, [ MoO_4_^2-^]=0 mM，[Hg^2+^]=500 ng/L, O_2_ Concentration (0%) |
|  |  | DE-Hg500-Mo0.25-0 | Inoculate with DE bacteria, [ MoO_4_^2-^]=0.25 mM，[Hg^2+^]=500 ng/L, O_2_ Concentration (0%) |
|  |  | DE-Hg500-Mo0.5-0 | Inoculate with DE bacteria, [ MoO_4_^2-^]=0.5 mM，[Hg^2+^]=500 ng/L, O_2_ Concentration (0%) |

Table S3 Description of transcriptome sequencing sample

| Treatments | Note of Treatments |
| --- | --- |
| 3h-Hg0 | Inoculate with B3 bacteria, [Hg^2+^]=0 ng/L, O_2_ Conc.(21%), Samples were collected at 3h of culture |
| 3h-Hg500 | Inoculate with B3 bacteria, [Hg^2+^]=500 ng/L, O_2_ Conc.(21%), Samples were collected at 3h of culture |
| 9h-Hg0 | Inoculate with B3 bacteria, [Hg^2+^]=0 ng/L, O_2_ Conc.(21%), Samples were collected at 9h of culture |
| 9h-Hg500 | Inoculate with B3 bacteria, [Hg^2+^]=500 ng/L, O_2_ Conc.(21%), Samples were collected at 9h of culture |
| 24h-Hg0 | Inoculate with B3 bacteria, [Hg^2+^]=0 ng/L, O_2_ Conc.(21%), Samples were collected at 12h of culture |
| 24h-Hg500 | Inoculate with B3 bacteria, [Hg^2+^]=500 ng/L, O_2_ Conc.(21%), Samples were collected at 12h of culture |
